# Blockchain-Based Decentralized Digital Manufacturing and Supply for COVID-19 Medical Devices and Supplies

**DOI:** 10.1109/ACCESS.2021.3118085

**Published:** 2021-10-05

**Authors:** Walaa Alkhader, Khaled Salah, Andrei Sleptchenko, Raja Jayaraman, Ibrar Yaqoob, Mohammed Omar

**Affiliations:** Department of Industrial and Systems EngineeringKhalifa University of Science and Technology 105955 Abu Dhabi United Arab Emirates; Department of Electrical Engineering and Computer ScienceKhalifa University of Science and Technology 105955 Abu Dhabi United Arab Emirates

**Keywords:** Blockchain, ethereum, decentralized digital manufacturing, supply chain, security, traceability

## Abstract

Coronavirus 2019 (COVID-19) has disclosed the deficiencies and limitations of the existing manufacturing and supply chain systems used for medical devices and supplies. It enforces the necessity to accelerate the shift towards decentralized digital manufacturing and supply chain networks. This paper proposes a blockchain-based solution for decentralized digital manufacturing of medical devices and their supply. We develop Ethereum smart contracts to govern and track transactions in a decentralized, transparent, traceable, auditable, trustworthy, and secure manner. This allows overcoming certain issues hindering the transition towards decentralized digital manufacturing and supply, including trusted traceability, attestations, certifications, and secured intellectual property (IP) rights. We incorporate the decentralized storage of the InterPlanetary file system (IPFS) into the Ethereum blockchain to store and fetch Internet of things (IoT)-based devices records and additional manufacturing and supply details. We present the system architecture and algorithms along with their full implementation and testing details. Furthermore, we present cost and security analyses to show that the proposed solution is cost-efficient and resilient against well-known vulnerabilities and security attacks. We make our smart contracts code publicly available on GitHub.

## I. Introduction

Coronavirus 2019 (COVID-19) is highly contagious and transferred through droplets or from touching surfaces carrying the virus. The relatively high death rates and exponentially increasing infection rates made COVID-19 a public health crisis. Governments worldwide imposed precautionary regulations in response to the pandemic’s rapid spread, including social distancing, lockdowns, and land and air commute ban with complete borders shutdowns. In addition, the global economic system has been severely affected by the COVID-19 disease. The pandemic has posed numerous new challenges that require a rapid response. For instance, it has increased pressure on the global manufacturing and supply systems. In addition, the high demands for medical supplies and devices resulted in a shortage of swabs, masks, test boxes, personal protective equipment, and body containment units. This massive demand had led to several operational issues.

In recent years, the demand for health and medical supplies has unprecedentedly increased globally. The production using traditional manufacturing techniques has been slow down due to lockdowns and disruptions. It has caused the demand for geographically scattered, small, agile, and flexible manufacturing workshops. The recent advancements in technologies, automation, digital manufacturing, Internet of things (IoT) devices, and the security and authenticity of data exchange made it possible to overcome this need. Below are given the main challenges faced by the existing centralized manufacturing and supply chain processes due to the COVID-19 pandemic:

• Centralization of production and the need to transfer manufactured products from production centers to geographically remote places.
• Extended lead times due to lockdowns, border closure, and long and thorough safety procedures for decontamination of transferred goods.
• Increased demand for urgently needed medical devices and supplies that the existing manufacturing facilities have not met.
• The lack of manufacturing/production flexibility constrains the shift of production lines among different product genres.
• Authenticity and traceability of medical devices and supplies produced in new and emerging facilities.

^1^https://github.com/WalaKhader/BC-DDM-COv19-Med/blob/main/Solidity-Codes

Recent advancements in automation, IoT, and cyber-physical systems have made today’s decentralized digital manufacturing operations much easier and manageable. They enable to tackle the sudden peaks in demand while maintaining the imposed social distancing and government regulations. Trends toward “glocalization” [1] can be carried using digital manufacturing technologies (3D printing, robotics, and CNC Machining) with the aid of online platforms. The medical industries are continuously growing the additive manufacturing (AM) applications, such as medical supplies to bone tissues engineering medical implants with customized design.

**FIGURE 1.**
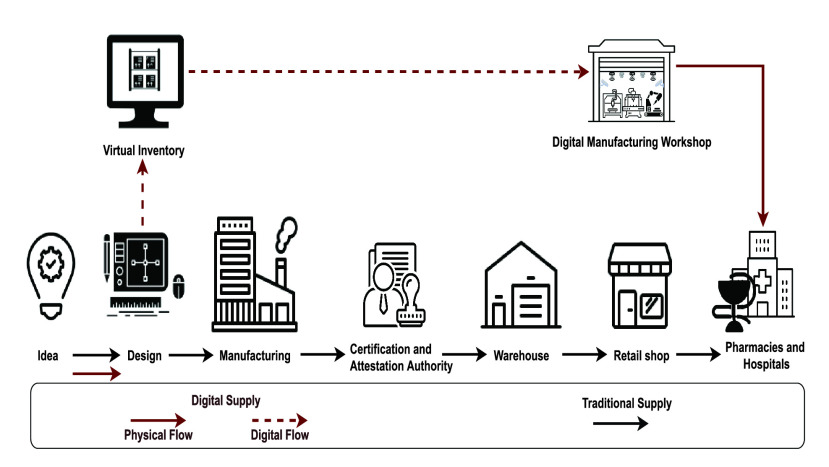
Traditional v.s. decentralized digital manufacturing and supply systems.

Decentralized digital manufacturing enables to overcome the challenges imposed by the COVID-19 pandemic on the traditional manufacturing and supply systems considering their multiple benefits, such as enabling shorter lead times, reducing transportation and inventory costs as shown in figure 1, customization, and increasing production flexibility. However, attestation, certification, and IP rights are some of the issues that still need to be addressed.

Medical devices are subjected to strict certification processes that vary depending on each country’s healthcare regulations and approvals. This adds to the hurdles in terms of required urgent supply and delivery of medical devices and supplies. The extensive and lengthy testing procedures and the long distances that threaten traceability, fast response, security, and trust can be achieved through blockchain-based decentralized digital manufacturing. The concept of blockchain technology has been introduced in 2008 [2]. Blockchain offers distinctive features, such as decentralization, audit, immutability, traceability, security, and trust [3]. It is governed by a group of computer nodes named clusters that work together on verifying and executing transactions. The technology uses cryptography (hashes) and digital signatures, for which two keys are utilized, public and private—generated from the Ethereum address and mining following the consensus mechanism. Ethereum smart contracts enabled decentralized applications are developed using the Solidity language. The proposed Ethereum blockchain-based solution is embedded both in on-chain recording the transactions and off-chain storage for documentation.

Although decentralized digital manufacturing can contribute in advancing automation, there are some challenges related to the attestation and certification of the product, traceability of the product throughout the development and supply chain process, and protection of IP rights, hindering its widespread adoption. In this paper, we aim to address these challenges.

### A. Related Works

Herein, we discuss related studies conducted on AM and digital manufacturing to deal with the COVID-19 pandemic. Also, we explore the existing blockchain-based solutions proposed to ensure traceability and authentication of the medical devices and supplies during the pandemic.

The study conducted in [4] shows that 3D printing technologies proved the capability of AM as a mass-production platform that is cost-efficient and reliable for medical devices. One of the main challenges faced by AM production during the COVID-19 pandemic is related to regulations of medical devices and acquiring approvals from official regulatory authorities [5]. However, federal agencies and governments have imposed regulations for 3D-printed medical products to follow and abide by them. An international risk classification system is made available for additively manufactured medical equipment and supplies [6]. The intellectual property (IP) and legal ownership issues were another main concern by authorities and medical institutes and clinics using those 3D printed parts. From a technical perspective, it is required to incorporate pre-processing and post-processing into AM manufacturing to minimize users’ intervention. Also, manufacturers can simulate printing before initiating the process using simulation software to forecast the microstructural alterations and the potential final status of the final part [7].

The authors in [8] proposed a workflow that enhances AM production of medical ventilation systems. They also emphasized the need to conduct a thorough assessment and analysis before selecting materials and technologies. COVID-19 has strained healthcare resources resulting in a shortage in several medical devices, including personal protective equipment (PPE), artificial respirators or ventilators, isolation chambers [9], and COVID-19 testing swabs kits. The shortage in swab kits is an explicit limitation to accurately identifying infected people [10].

The authors in [11] discussed several types of polymers that can be used in 3D printing, such as PE, PA, PC, PVC, PU, PLA, and Silicone. They also explain the testing and validation required to approve the use of these materials. The material properties like fatigue and failure in performance are specified and determined by International Standards Organization (ISO), United Laboratories (UL), and the American Society for Testing and Materials (ASTM) standards. The Food and Drug Administration (FDA) reviews and approves the device/product for manufacturing and marketing when it is safe for public use. The research also clarifies that testing and acquiring approvals and government acceptance is one of the main challenges for AM-produced parts.

In [12], the authors highlighted how the pandemic raised the use of AM because of its flexibility and capability to fulfill the growing demand. They also explain the importance of shifting toward a digital supply chain during a similar crisis. The system’s elements include the digital inventory where all digital designs can be shared worldwide, distributed digital manufacturing hubs, raw material, and local transportation to hospitals’ locations. The authors in [13] implemented text mining and machine learning algorithms to analyze online content on AM and COVID-19. They concluded that AM is primarily used to manufacture medical devices and parts. They also discuss the necessity to standardize 3D printed parts and establish specific digital manufacturing certification procedures.

The researchers in [14] pointed out the benefits of incorporating digital supply and digital manufacturing to overcome the obstructions and deficiencies straining the medical sector. They further discuss case studies of Air-Purified Respiratory Hood, COVID-19 specimen collection kits, ventilator venturi valve, facemasks, modified surgery helmet [15], ventilator nozzles, 3D Printed Ventilator Circuit Splitter, and 3D Printed Artificial Lungs. Some of the gains discussed include the direct and agile digital manufacturing of customized parts, minimized waste of material, which ensures better utilization of available material. Also enabling decentralized manufacturing and cutting downtime, increased accessibility, localized manufacturing [16], and minimized direct human intervention required, which fits perfectly with the current pandemic conditions [17].

In [18], the authors analyzed different technologies of AM, including fused filament fabrication (FFF) and selective laser sintering (SLS) technology. The results reveal that the used polymers’ mechanical and structural stability is not affected by the disinfection, and the printed parts can be reused, featuring the quality of AM-produced parts. Fused Deposition Modeling (FDM) was implemented successfully to print novel faceshields [19].SLA can be used for test swab printing and lung models for surgical purposes [20] and Multi-jet fusion (MJF) for face masks and door openers [21]. Furthermore, Wierzbicki *et al.* [22] implemented AM for producing PPE face shields and proved in their work AM ability to allow mass production in a short time and help in reducing disruption caused by the shortage of medical supplies.

Tarfaoui *et al.* [23] identified the fundamental weaknesses and threats of AM in the medical fields, such as lack of sufficient expertise needed, post-processing, material limitation, copyrights, and IP issues, regulations, and dangerous weapons and security challenges. Furthermore, they highlighted the advantages and opportunities, including time efficiency, sustainability, minimized cost of transportation and inventory, the flexibility of design and enabling complex shapes production, decentralization of manufacturing, allowing make-to-demand, customization, and new product development. To obtain fully decentralized supply systems, the cooperation of researchers from various backgrounds, institutes, and authorities is required [24]. The authors in [25] provided recommendations to enhance the system’s resilience in the future, including establishing a pre-validated and approved virtual library of design files to be used in emergency cases along with clear regulations, procedures, and standards provided by the relevant authority.

The COVID-19 pandemic imposed new regulations and living conditions that forced decentralized solutions using the Internet-based platforms for most human lives aspects. Specific challenges, such as security, traceability, authentication, and trust, still need to be addressed. Blockchain can help to overcome such challenges. Several studies proposed blockchain-based solutions enabling the decentralized, secure, and immutable flow of information and data among different institutes and parties to handle COVID-19 related issues efficiently. The authors in [26] proposed an Ethereum blockchain supply chain and waste management solution for medical devices during the recent pandemic. The proposed solution aims to assist authorities in ensuring that the medical wastes are disposed of properly. The proposed system is cost-efficient and reflective of the blockchain attributes, including traceability, audibility, security, and reliability. Unlike this work, our study mainly focuses on the manufacturing aspect of medical supplies instead of its waste disposal.

The authors in [27] proposed an Ethereum blockchain-based solution to track the supply chain of PPE products during the COVID-19 pandemic. The cost and security analyses revealed the effectiveness of the system. Another blockchain-based solution has been proposed in [28] for creating COVID-19 digital medical passports and immunity certificates to prevent the spread of COVID-19. It also enables authorities to detect potential threats accurately and identify immune individuals in a secure, traceable, immutable, and reliable manner. The solution employs Ethereum smart contracts and re-encryption proxies. The results reveal that the proposed solution is cost-efficient and tamper-proof.

Strengths of the proposed use of blockchain include enabling automation, immutability, trust, transparency, and decentralization [32] at relatively low costs [31]. On the other hand, one threat could be the lack of sufficient expertise and the resistance to change [33]. The key benefits of implementing blockchain technology and other technologies (i.e., machine learning, big data, and cloud computing) to combat the COVID-19 pandemic in terms of the medical supply chain, clinical trial management privacy protection, and donation have been discussed in [29], [30], [34].

Table 1 summarizes the existing blockchain-based frameworks proposed to solve medical and healthcare-related issues during the COVID-19 pandemic. To the best of our knowledge, none of the existing studies proposed a blockchain-based digital decentralized manufacturing solution to address the rising hurdles in the manufacturing of medical devices and supplies during the pandemic.

**TABLE 1:** Blockchain and COVID-19 Related Research Work

Publication	Similarity in Targeted Problem	Framework	Implementation of SCs	Number of SCs	Participants
Ahmad Raja et al [26] Forward Supply Chain and Waste Management	Slightly similar (traceability of data in traditional SC)	Provided	Successful (Remix IDE)	Ethereum SCs (4)	Manufacturer, Distributor, Hospital, Waste shipper, FDA and Waste treatment facility
Ilhaam Omar et al [27] Traceability for COVID-19 PPE	Slightly similar (traceability of products in traditional SC)	Provided	Successful (Remix IDE)	Ethereum SCs (3)	FDA, Manufacture, Distributor, Wholesaler, Provider
Haya R. Hasan et al [28] Digital Medical Passports and Immunity Certificates	Not related	Provided	Successful (Remix IDE)	Ethereum SCs (4)	Globally approved MoHs, MoFA, Approved Testing Centers, Patients
Sharma et al [29] Digital data storage of COVID-19 patients, Vaccine tracking, pandemic locations detection and donations	Not related	Provided	None	None	Patients, hospitals, testing labs, governments
Dounia Marbouh et al [30] Clinical trial management,medical supply chain, outbreak tracking and donation tracking using oracles	Slightly similar (traceability of products in traditional SC)	Provided	Successful (Remix IDE)	Ethereum SCs (3)	DAPPs and dashboards, Oracles and web feed sources.
Min Chang et al [31] Role of Blockchain to fight against COVID-19	Slightly similar (traceability of data in traditional SC)	Not Provided	None	None	NA
Anjum Khurshid [32] Crisis of supply chain trust during the COVID-19 Pandemic	Slightly similar (traceability of products in traditional SC)	Provided	None	None	Manufacturers, wholesalers, governments, pharmacies and patients
Antonio Fusco [33] Proposing a predictive system with Blockchain and AI to contain pandemic risks	Not Related	Provided	None	None	Governments, citizens,researchers and healthcare operators
Our Proposed Solution Blockchain-based Decentralized Digital Manufacturing for medical devices	–	Provided	Successful (Remix IDE)	Ethereum SCs (4)	FDA, researchers, hospitals, MoH, Designers, Digital workshops and Virtual Inventory

### B. Contributions

Unlike the aforementioned studies, this paper proposes a blockchain-based solution for attestation and certification, tracking, and securing IP rights of digitally manufactured medical devices and supplies. The main contributions of this paper are as follows:

• We propose a blockchain-based solution to enable decentralized digital manufacturing to produce medical devices and supplies in a transparent, traceable, auditable, trustworthy, and secure manner. Also, it assists in supply chain management.
• We develop Ethereum smart contracts to establish the legitimacy of the digitally manufactured medical products through enabling certification from geographically distant locations relying on the secured and trusted traceability of medical products and supplies. We incorporate the Interplanetary file system (IPFS) decentralized storage into the Ethereum blockchain to manage the large-size data.
• We present the system architecture, sequence diagrams among participants, and algorithms describing the functions of the smart contracts that control the interactions between participants. We provide their full implementation details along with the smart contracts’ code that is made publicly available on the Github repository^1^.
• We evaluate the proposed approach using cost and security parameters. Also, we compare our solution with the existing solutions to shows its novelty. Our proposed approach is generic and can be adapted into various emergency use case scenarios.

The rest of the paper is organized as follows. Section II discusses our proposed solution. Section III presents the implementation details. We provide testing and validation details in Section IV. We evaluate the proposed solution in Section V. Finally. We conclude the paper in Section VI.

## II. Proposed Blockchain-Based Solution

This section presents the proposed decentralized blockchain-based digital manufacturing and supply chain solution to control and provide medical devices and supplies during the COVID-19 pandemic. In the following, we describe the system stakeholders, components, interactions, and implementation details in detail.

### A. System Participants and Components

Herein, we describe each participant’s role in the decentralized digital manufacturing blockchain-based supply network to meet high demands due to COVID-19 in medical equipment and devices. We also discuss key components of the system.

#### 1) Medical Supplies and Equipment Designers

They are entitled to design products and create their digital copy (blueprint). They specify details like size, dimensions, structure analysis, and details, such as the material, printing technology, and printing environment parameters, including temperature, pressure, and humidity.

#### 2) Research Institutes (RIs)

They are responsible for sharing their state-of-the-art studies, technologies, and materials to improve the printing process, parts characteristics, quality, time of printing, and all related aspects. Their role is most significant in crisis times, such as the COVID-19 pandemic, where new and unusual practices emerge like social distancing and border closure. On the other hand, it is critical for new and novel products.

#### 3) Virtual Inventory

It is a cloud database owned by the Ministry of Health (MoH) and created by their registration entity. It contains all digital copies (blueprints) of approved designs by the FDA that have been successfully printed and used at least once upon agreements made with their designers. The virtual inventory’s role is to substitute physical inventory and reduce its costs. Also, in crisis times, such as the COVID-19 pandemic, when a rapid and flexible response is vital, the virtual inventory facilitates and increases the speed of response. As the hospitals and clinics need certain parts existing on the virtual inventory, they can skip the entire design phase of the supply process and move directly to the manufacturing phase in nearby workshops.

#### 4) Digital Manufacturing Workshops

It is a distributed network of workshops geographically scattered to shorten distances and reduce the time of delivering the product after production to the end-user. Each workshop is digitally enabled with the machines (3D printers, CNC machines, and Robots), in addition to the IoT devices. Finally, each workshop has its raw material stock that meets specifications put by the designer and FDA.

#### 5) IoT Sensors and Cameras

They are used to record the manufacturing process in the workshop and all the surrounding environment measures like temperature, pressure, vibration, and any machine-related metric.

#### 6) Hospitals and Medical Clinics

They represent the end-user in this system, and they initiate the flow of interactions when they have a shortage by asking for medical supplies and products, specifying parts and quantities needed.

#### 7) Certification Authority (FDA)

It is responsible for setting standards and procedures, authorizing and certifying materials and products based on the data retrieved from IoT devices, designs blueprints, procedures, and specifications provided by the designer and the workshops.

#### 8) MoH Registration Entity

It is responsible for registering designs after agreements with designers authorized and certified by the FDA after completing at least one successful supply process. Then, the entity assists in adding the new approved designs (blueprints) to the virtual inventory and granting hospitals and medical clinics the authority to access this virtual inventory and the accompanying list of registered digital manufacturing workshops.

#### 9) IPFS

It assists in storing large-size files on the blockchain ledger. It helps to manage blueprints, IoT device records, data related to tests, technologies details, and specifications uploaded by designers, and digitally-enabled workshops used by the certification and attestation authority to make decisions certifying parts materials, technologies, and procedures. The file locations in the IPFS are identified using hashes that are transferred through smart contracts to the authority and to stored on the blockchain ledger.

#### 10) Smart Contracts

They are programs constituting a set of functions written in a programming language and work as an intermediate among all stakeholders to govern and record their interactions on the blockchain ledger. In this research, Ethereum smart contracts are used and written in the solidity programming language. We develop two smart contracts. The former is used to govern the process in both phases (design and production). The latter is used to register designs, parts, designers, and workshops and their approval certificates and confirm the upload of newly approved blueprints to the virtual inventory of MoH.

### B. System Architecture

**FIGURE 2.**
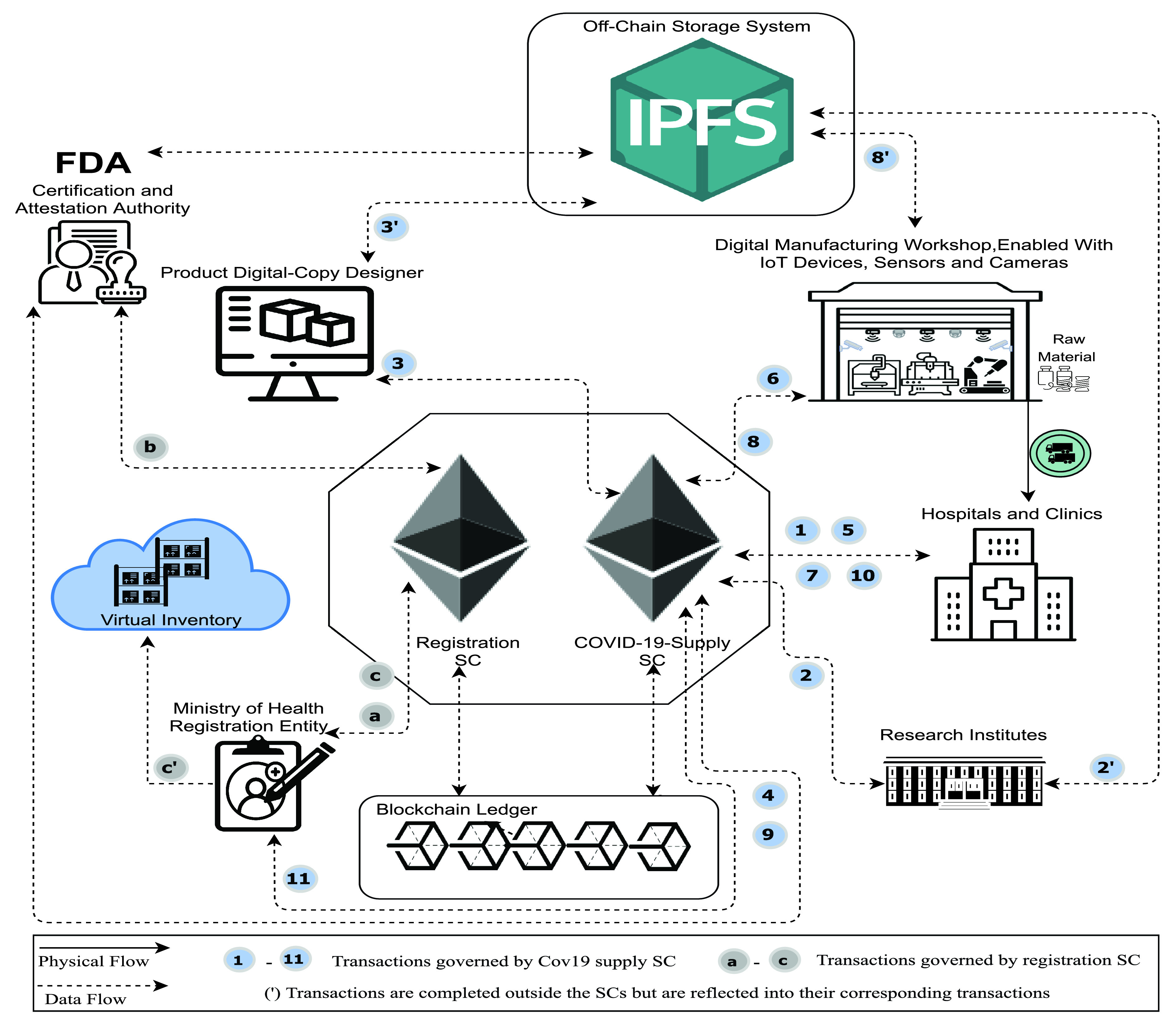
System architecture showing the main components, participants, and interactions among the different system elements for new designs and products.

As demonstrated in Figure 2, the manufacturing process can be divided into two phases (design and manufacturing). When a hospital faces a shortage in some medical devices or supplies, they first check the virtual inventory, and if the product design (blueprint) does not exist on the virtual inventory, the first alternative of full design and manufacturing supply is followed. The design phase is necessary for new products and new designs that do not exist on the virtual inventory. Figure 2 illustrates all participants and their interactions. The design phase is initiated when the hospital or clinic requires a design for needed equipment or supplies. RIs provide their state-of-the-art technologies and materials, the designer bids to design, and once approved by the authority and end-user, we move into the second phase. Digital manufacturers bid to produce the approved design, and when approved, the manufacturer starts production. After getting the final approval, the manufacturer shares the data, test details, IoT device recordings, and others. Once final approval is acquired, the products are delivered and successfully operated to register the supply participants, and the new design is forwarded to the registration entity.

The registration entity initiates the $SC_{REG}$. It receives the supply process logs and participants; then, it verifies the certificates and approvals with the certification authority. Once verified, the new design (blueprint) is uploaded to the virtual inventory for future uses.

**FIGURE 3.**
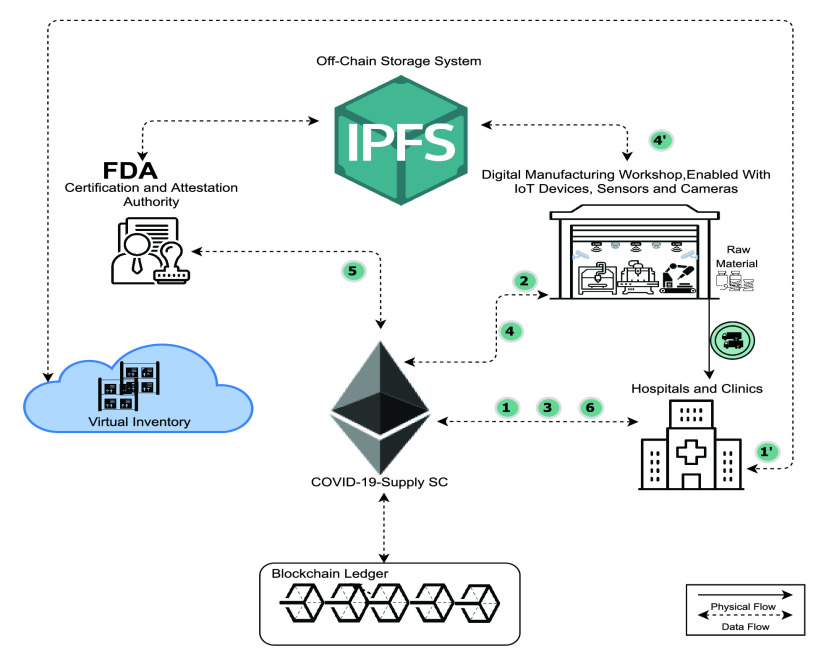
System architecture showing main components and the interactions among the system elements for designs existing on the VI.

Figure 3 illustrates the use of the system for products already existing on the virtual inventory. This demonstrates the critical role of the virtual inventory in shortening the supply process. Once the design is available, the hospital or end-user can skip directly to the second manufacturing phase.

## III. Implementation Details

Herein, we show the implementation of our developed smart contracts along with algorithm details. Our proposed system includes two smart contracts, namely, the COV19-SUPPLY-SC and $SC_{REG}$ for medical devices, tools, and supplies during the pandemic. The MoH creates smart contracts. Each participant is assigned a unique ID to trace and govern the complete process of hospitals and clinics declaring shortage, RIs specifying technology and material, designing, digitally manufacturing a product until final product delivery to hospitals and clinics, and the registration process completion governed by the registration smart contract. Solidity language is used to write smart contracts. We employ Remix IDE for compilation.

### A. COVID-19 Medical Parts Supply Smart Contract

The process is initiated when a hospital or clinic declares a shortage in a medical device, tool, or supplies. At this point, they first check the virtual inventory of MOH. Our smart contract announces that need through an event if the product is not available on the virtual inventory or existing models do not fulfill a new emerging need. RIs share their latest and most relevant research and suggested feasible technologies, manufacturing environmental conditions, and materials using an IPFS hash through the COV19-SUPPLY-SC. The IPFS hash is saved on the chain. At the same time, the actual records and documents are saved and uploaded off the chain on the IPFS. Based on the RIs input, designers bid with their digital designs; then, an event is emitted requesting approval of the design. The certification authority reviews the designs and structure simulations once approved stakeholders are notified with an event. The hospital or end-user approves the design and asks for a digital manufacturing offer.

This point marks the beginning of the second phase: the manufacturing phase; the hospital or clinic can skip the first phase if the product meeting their specific needs is available on the virtual inventory. The hospital directly asks for printing offers to replace the designer ID with the virtual Inventory ID. After that, an event is triggered to announce the request for manufacturing, and digital manufacturers provide their bids and ask for bid approval. The hospital accepts the best offer and asks to initiate digital manufacturing. The workshop receives a notification and starts manufacturing the medical devices or supplies.

**FIGURE 4.**
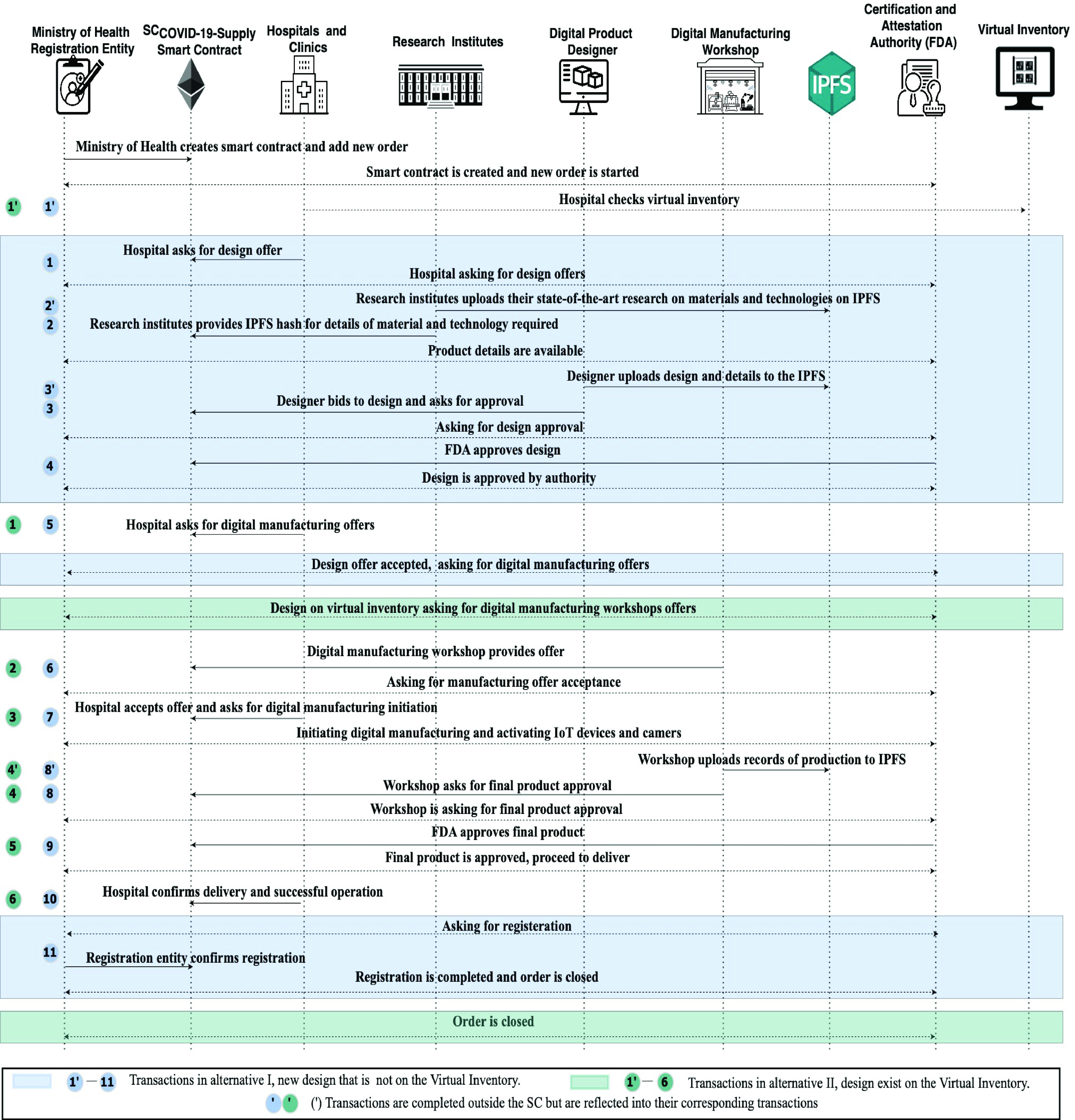
Sequence diagram showing the interactions among participants in the COV19-SUPPLY-SC.

After finalizing the production, the digital manufacturing workshop asks for the final product and manufacturing process approval from the authority (FDA) and the hospital. Once it is approved, the product is delivered to the hospital through local delivery. The hospital confirms delivery and proper operation of the final product. Also, it asks to register the order and add the design to MOH’s virtual inventory if it is a new design. Once registration is confirmed, all stakeholders are notified of an event. Figure 4 presents the proposed decentralized Digital Manufacturing blockchain-based COVID-19 Supply system Sequence diagram.

In the following, we present the algorithms that describe different functions of the COV19-SUPPLY-SC.

#### 1) Hospital Asks for Design Offers

In Algorithm 1, the Hospital declares the shortage of a specific medical device or supply. The input required by the algorithm is the hospital ID and the product ID. The smart contract verifies if the caller’s ID matches the Hospital’s ID. and issue an event to request design offers.

**Algorithm 1:**
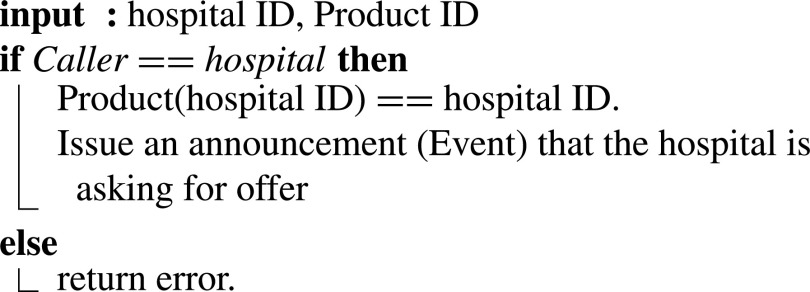
Hospital Asking for Design Offers

#### 2) RIs Confirms Required Manufacturing Details

RIs are notified through the previous algorithm that hospitals and clinics face a shortage of medical devices or supplies that do not exist on the virtual inventory. Using their state-of-the-art research and algorithm 2, they provide details of the most feasible and suitable technology, manufacturing environment parameters, and material to be used. All the relevant data is stored off-chain on IPFS (task 2’ in figure 4), and the hash is shared through the COVID-19 supply smart contract. The input values include RI’s ID, product ID, and the RI-IPFS Hash. The function verifies the RI’s identity and adds the manufacturing specifications’ IPFS hash, and an event is emitted announcing that product manufacturing details are available.

**Algorithm 2:**
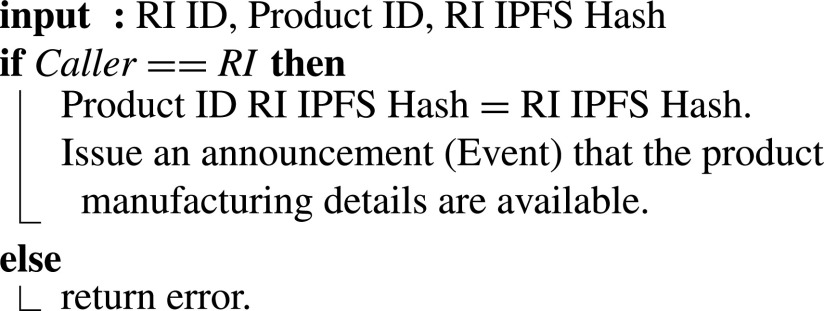
RIs Confirms Required Manufacturing Details

#### 3) Designer Asking for the Proposed Design Approval

Once the design is completed, the Designer puts the files and simulations on the IPFS and makes a hash used to access the uploaded files. Using algorithm 3, the Designer asks for approval from the certification authority. The input for this algorithm includes Design IPFS hash, Hospital ID, and product ID. The hash allows the certification authority to access all the needed data, check and examine then make their decisions. Algorithm 3 demonstrates how this function works.

**Algorithm 3:**
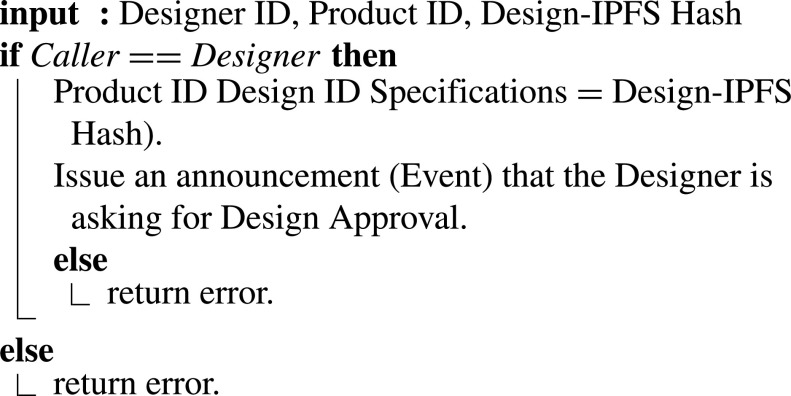
Designer Asking for the Proposed Design Approval

#### 4) Authority Design Approval

The smart contract takes the Authority ID, product ID, and Authority-decision as input parameters, as shown in algorithm 4 [35]. Design details on IPFS can be accessed by the authority using the design hash announced in the preceding function. After reviewing the files and data provided and measuring the fit of the design based on the standards, the authority approves or rejects the design, and an event announces the decision to all participants either way.

**Algorithm 4:**
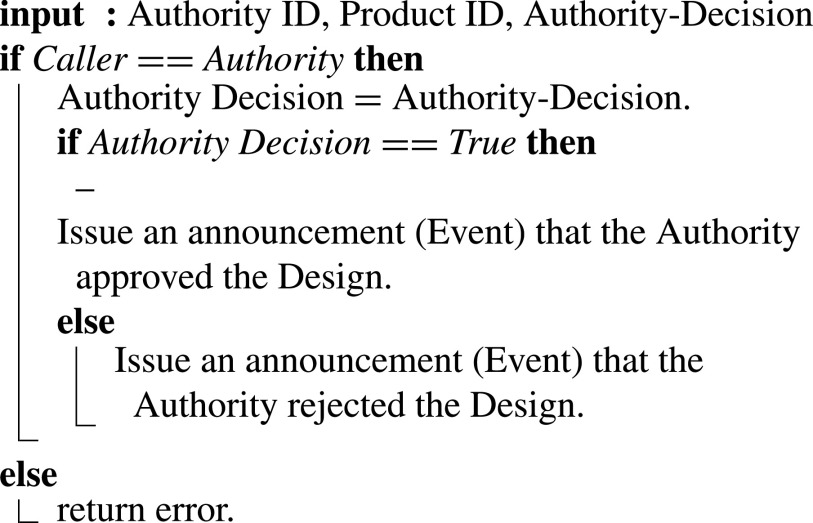
Authority Design Approval

#### 5) Asking for Digital Manufacturing Offers

In Algorithm 5 if the hospital accepts the design, an event is emitted announcing the need for manufacturing the design. Digital manufacturing workshops can submit offers to manufacture and deliver the medical devices and supplies, while if the hospital rejects, an event is triggered accordingly.

**Algorithm 5:**
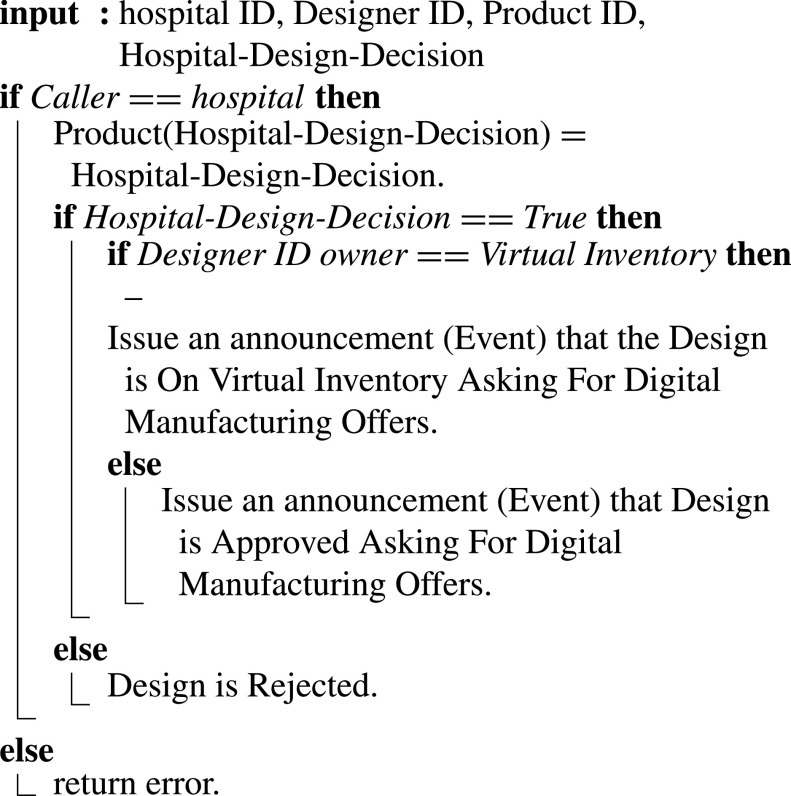
Asking for Digital Manufacturing Offers

The alternative scenario is if the design exists on the virtual inventory, then the hospital can skip the previous steps and start with this function by replacing the designer ID with the Virtual Inventory ID. Input are Designer ID, Product ID, Hospital ID, and Hospital decision on design. The smart contract verifies the hospital’s identity and records the hospital’s decision on the design.

#### 6) Workshop Offering Digital Manufacturing

Once the design is approved, digital manufacturing workshops receive the event announcement and provide their bids. The smart contract emits an event as per Algorithm 6. Digital Workshop ID, Product ID, and Price are the required inputs [35]. Detailed offers, including location, time to manufacture, time to delivery, and other relevant specifications, can be shared directly between the hospital and the digital manufacturing workshop. The hospital uses those details to select the most reasonable offer given how critical delivery time is for medical devices, in addition to the price.

**Algorithm 6:**
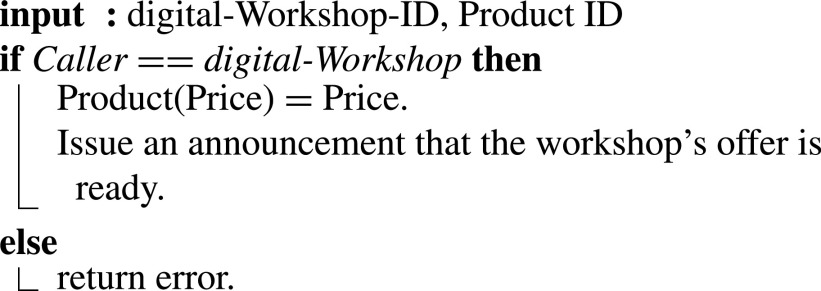
Workshop Offering Digital Manufacturing

#### 7) Hospital Accepts/Rejects Manufacturing Offer

In algorithm 7, the hospital accepts or rejects digital manufacturing workshop’s offer [35]. The smart contract ensures that the function’s caller is the hospital. If the hospital approves, an event is triggered to announce the hospital’s acceptance of the offer. Otherwise, a rejection notification is issued if rejected. The hospital might receive several offers from different digital manufacturing workshops, and depending on the offer details, the hospital makes its decision. The digital manufacturing workshop is ordered to commence digital manufacturing supported by IoT devices and process monitoring cameras.

**Algorithm 7:**
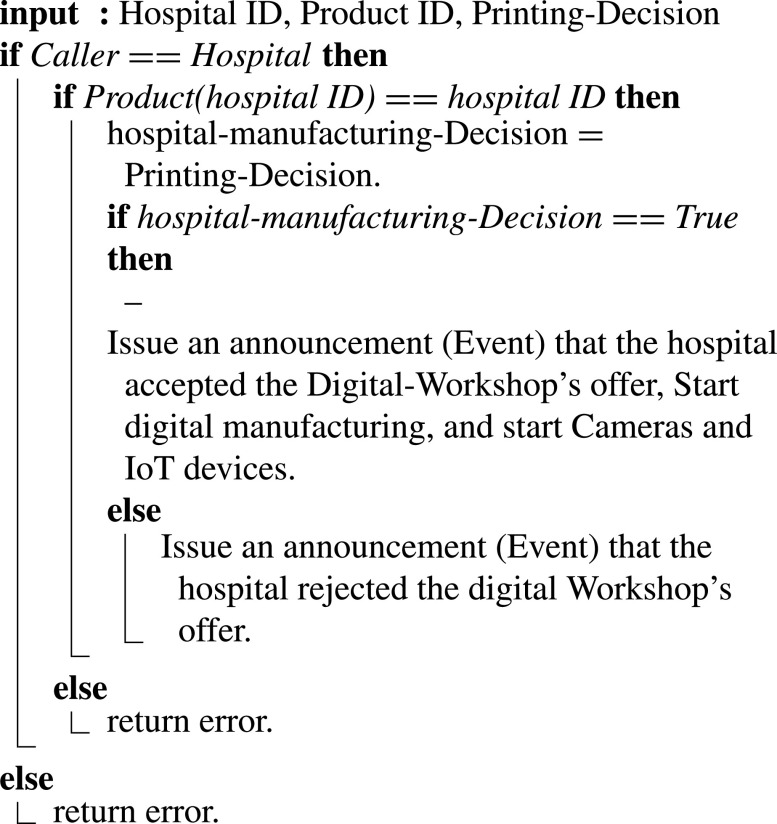
Hospital Accepts/Rejects Manufacturing Offer

#### 8) Requesting Approval of Certification Authority for the Finished Product

when manufacturing is completed, task (8’) is done by uploading all manufacturing records from IoT devices and cameras to an off-chain storage IPFS and generating a hash. The IPFS hash is provided through the smart contract function illustrated in algorithm 8 to the certification authority. Input to this function includes Digital-Workshop ID, Product ID, and Printing-IPFS hash. IoT devices and cameras records are to be used by the authority to validate the manufacturing process. IoT devices can enable real-time recording and transmission of data, and this adds more trust and reliability, reflecting more precise perception by the authority on the finished product.

**Algorithm 8:**
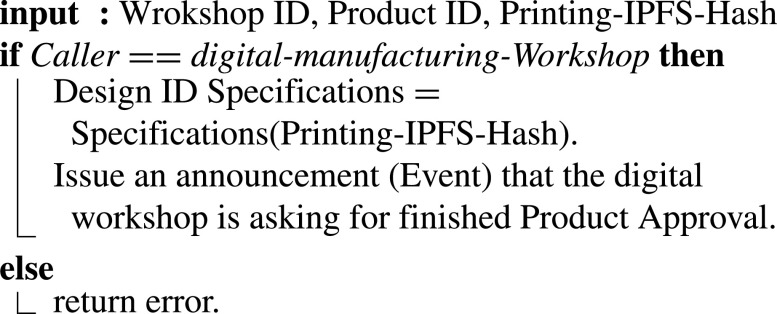
Requesting Approval of Certification Authority for the Finished Product

#### 9) Product Approval By Certification Authority

In algorithm 9, the authority approves/disapproves the manufactured product and the manufacturing process. The approval is announced through the smart contract, and delivery is triggered. Similarly, if rejected, the rejection notification is issued as well through the SC event.

**Algorithm 9:**
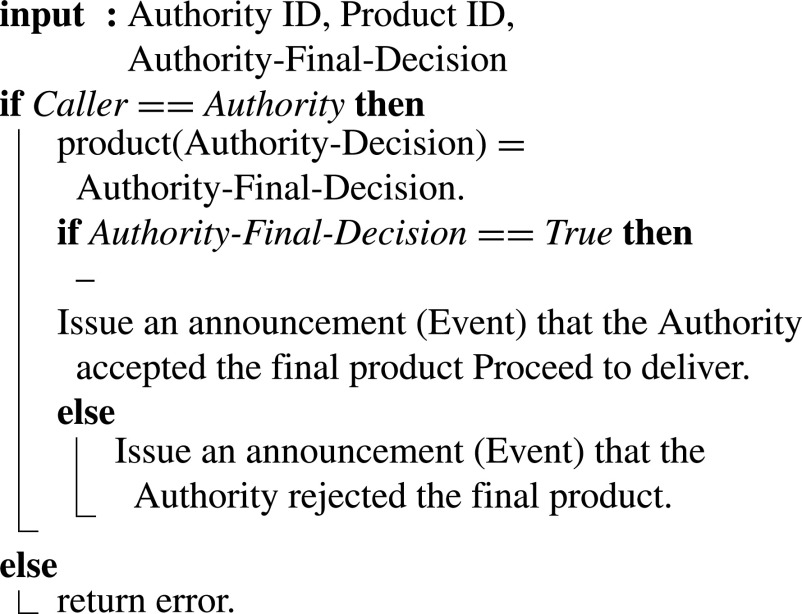
Authority Approves/Rejects Product

#### 10) Hospital Confirms Delivery and Successful Operation

Once the product is delivered and operated successfully, the Hospital confirms that, as in algorithm 10, inputs are hospital ID, Product ID, Designer ID, and Delivery Confirmation. If the product is received and is functioning correctly, the smart contract verifies whether or not the product’s design exists on the virtual inventory. If it is a new design, the smart contract announces the need to register this new design through an event. The registration is intended to be after the actual manufacturing and operation to ensure the reliability of the design before adding it to the MOH virtual inventory with at least one successful complete manufacturing and operation. Finally, if the product was not received or malfunctioned and not operating correctly, an announcement is made through a smart contract event informing all stakeholders.

**Algorithm 10:**
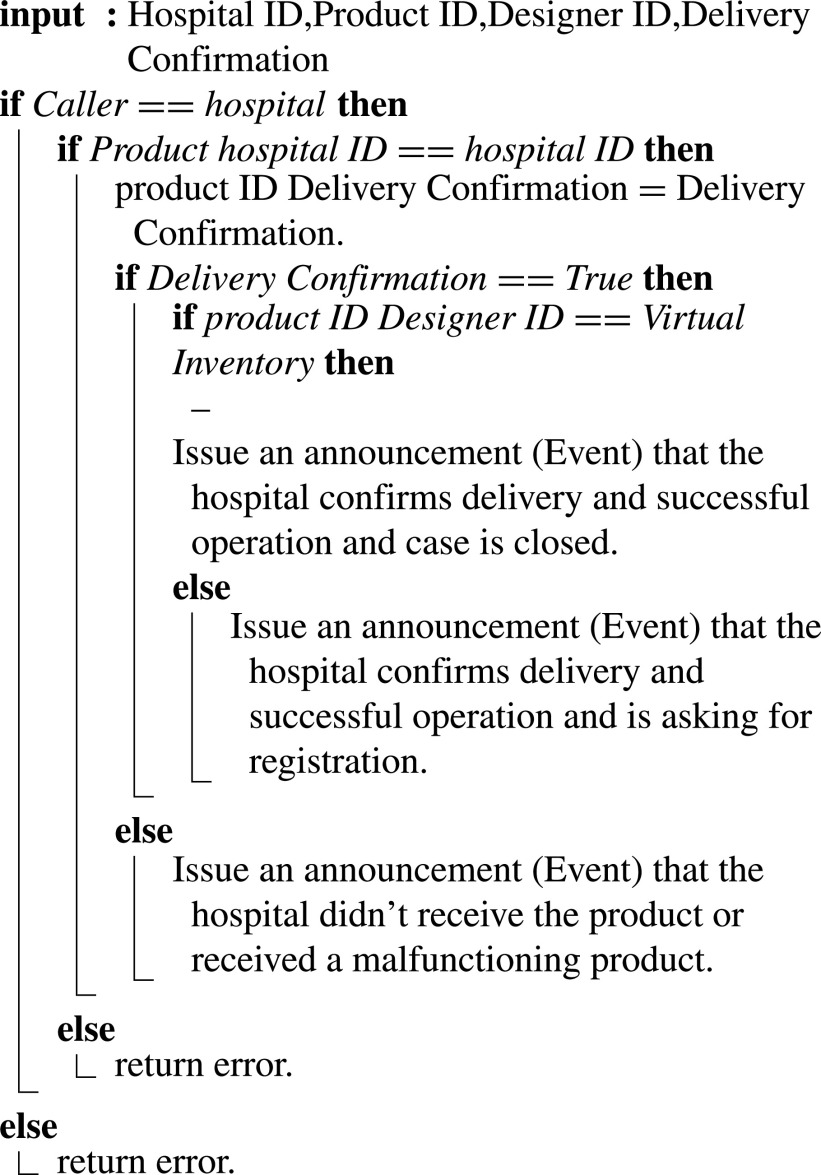
Hospital Confirms Delivery and Successful Operation

#### 11) MOH Confirming Registration and Virtual Inventory Update

As shown in algorithm 11, the registration is confirmed by the MoH. Inputs include Confirmation of Registration, MOH ID, Product ID. Once the registration is confirmed, the smart contract announces all stakeholders of the registration and addition of the new design to the virtual inventory.

**Algorithm 11:**
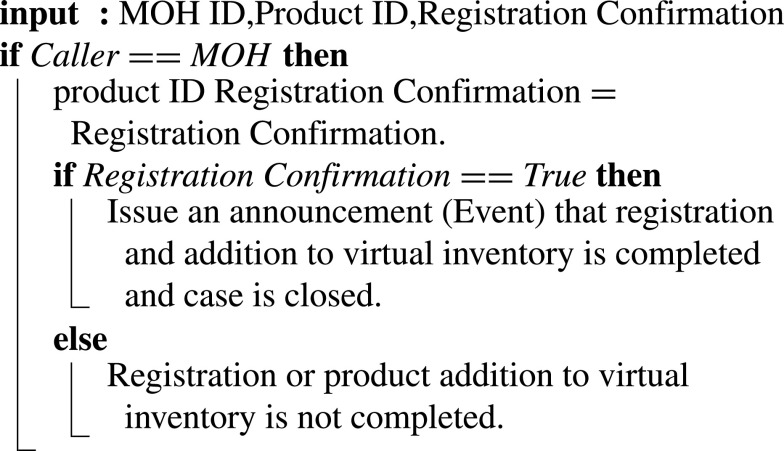
MOH Confirming Registration and Virtual Inventory Update

### B. Registration Smart Contract

**FIGURE 5.**
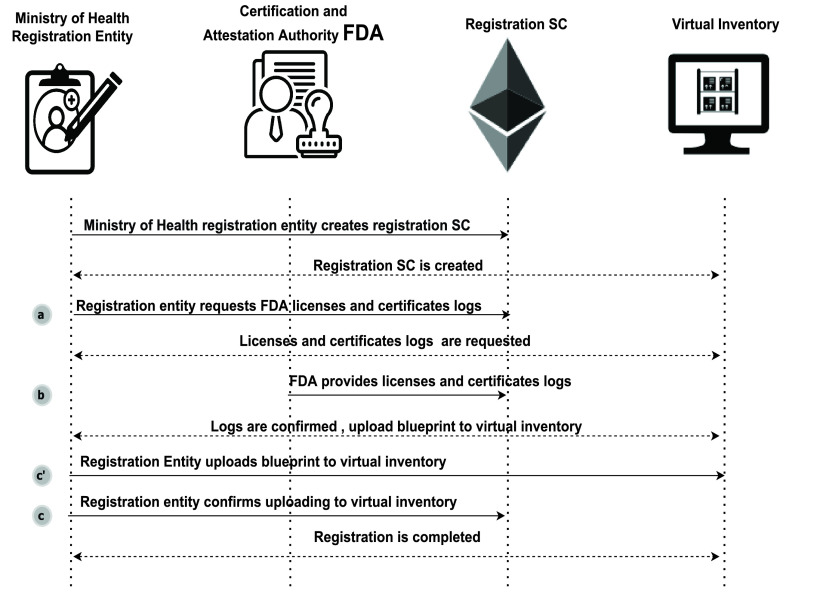
Sequence diagram of all interactions among participants with the registration smart contract.

The second smart contract helps to trace and control the registration process. The participants include the registration entity in MOH, Certification authority (FDA), registration smart contract, and the virtual inventory. As shown in figure 5, the process starts when the registration entity (MOH) requests FDA certificates and approvals numbers for all participants, designs, and final products. When the authority provides its logs and requested certificates and approvals numbers, adding the new design to virtual inventory is triggered. Upon agreements made with the designer, the blueprint (digital design) is added to the MOH virtual inventory, and when MOH confirms, the addition to VI registration is announced to be completed.

Hereafter we demonstrate the detailed algorithms representing functions of the $SC_{REG}$.

#### 1) MOH Registration Entity Requesting FDA Licences and Approvals

Algorithm 12 illustrates how the registration entity of the MOH verifies all licenses and approvals granted during the supply process governed by the supply smart contract by requesting the licenses and approval numbers, including hospital, designer, and digital manufacturer licenses. In addition to design records and manufacturing records approvals, the smart contract emits an event to the certification authority (FDA) to announce MOH’s need for license and approval numbers.

**Algorithm 12:**
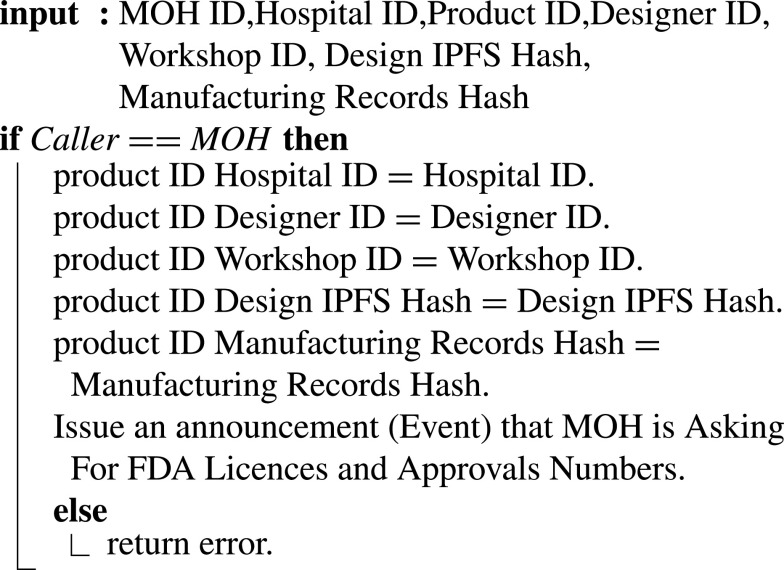
MOH Registration Entity Requesting FDA Licences and Approvals

#### 2) Certification Authority (FDA) Providing Licences and Approvals Numbers

Algorithm 13 shows how FDA responds with the approvals and license numbers through the smart contract. This step is to verify all participants and approvals before taking the initiative to add the new design to MOH’s virtual inventory. The smart contract announces the submission of logs requested to MOH using an event.

**Algorithm 13:**
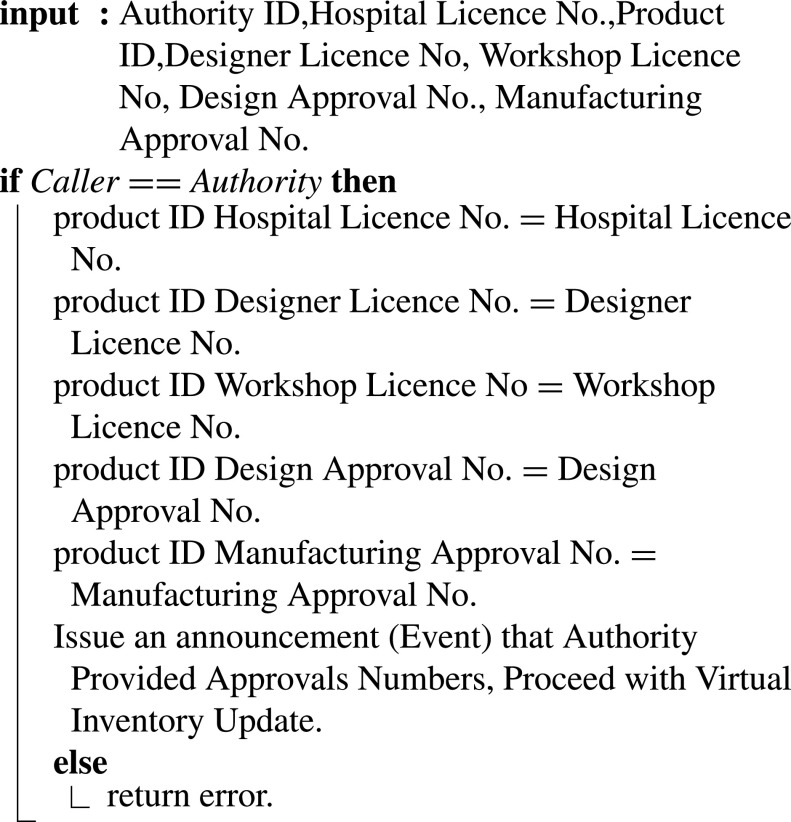
Certification Authority(FDA) Providing Licences and Approvals Numbers

#### 3) MOH Confirming Registration and Adding the New Design to the Virtual Inventory

Algorithm 14 illustrates how the MoH confirms the registration. Inputs include Confirmation of Registration, MOH ID, Product ID, and Virtual Inventory Address. Once the registration is confirmed, the smart contract triggers an event announcing that registration is completed and the new design (blueprint) is successfully added to the virtual inventory. Alternatively, an event is triggered to announce that the registration is incomplete. The addition of a design to the virtual inventory is first discussed with the designer, and once an agreement is made, the digital product or copy can be uploaded to MOH virtual inventory.

**Algorithm 14:**
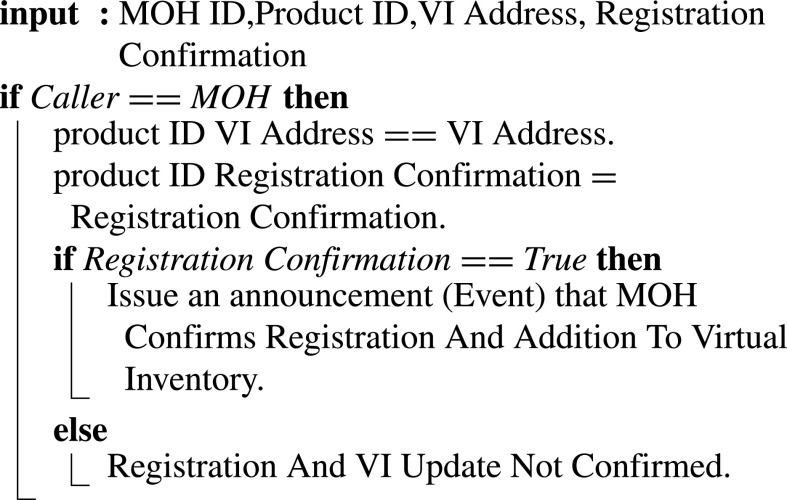
MOH Confirming Registration and Adding the New Design to the Virtual Inventory

## IV. System Testing and Validation

This section presents testing outcomes using Remix IDE, a desktop open-source application commonly used for developing, testing, and deploying smart contracts written in solidity language. We demonstrate the input, output, and log details for the developed smart contracts’ functions. Six participants are involved in our medical devices decentralized digital manufacturing testing scenarios. The participants that interact with the smart contracts are the registration entity (MOH), hospital, RI, designer, digital manufacturing workshop, certification authority (FDA), and a virtual inventory (VI). Table 2 shows the Ethereum addresses of the participants and the smart contracts.

**TABLE 2:** Participants in the Supply and Registration Smart Contracts and the Contracts Ethereum Addresses

User	ID	Ethereum Address
MOH	0	$0\times 5$B38Da6a701c568545dCfcB03FcB875f56beddC4
Hospital	1	0xAb8483F64d9C6d1EcF9b849Ae677dD3315835cb2
RI	2	$0\times 4$B20993Bc481177ec7E8f571ceCaE8A9e22C02db
Designer	3	$0\times 78731$D3Ca6b7E34aC0F824c42a7cC18A495cabaB
Workshop	4	$0\times 617$F2E2fD72FD9D5503197092aC168c91465E7f2
Authority	5	$0\times 17$F6AD8Ef982297579C203069C1DbfFE4348c372
VI	6	$0\times 5$c6B0f7Bf3E7ce046039Bd8FABdfD3f9F5021678
COV19-SUPPLY-SC	–	$0\times 7$EF2e0048f5bAeDe046f6BF797943daF4ED8CB47
$SC_{REG}$	–	$0\times 0$fC5025C764cE34df352757e82f7B5c4Df39A836

### A. COVID-19 Supply Smart Contract

All functions have input fields required or a condition that is tested successfully. The COV19-SUPPLY-SC’s functions can be divided into two phases (design and the digital manufacturing phase). For that, there exist similarities in the execution and outputs of some of the functions. Therefore, hereafter we share the results and logs of critical functions only, manifesting inputs and outputs of the smart contracts’ functions during the compilation due to the synergies among stakeholders and the smart contracts.

#### 1) Hospital is Asking for Design Offer

**FIGURE 6.**
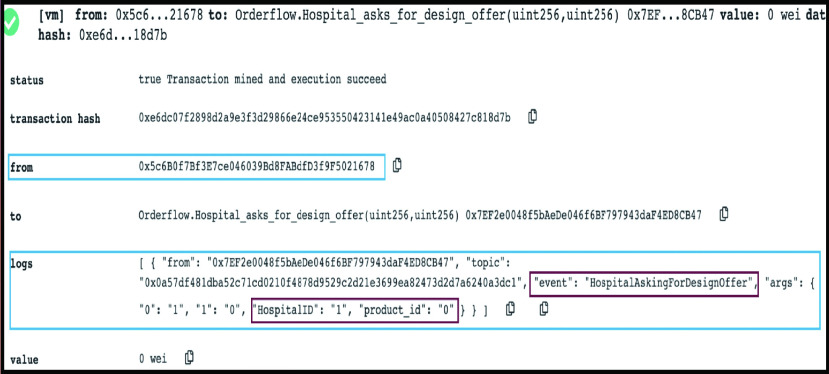
Logs showing that the hospital is asking for design offer.

After checking the virtual inventory and not finding the required product’s design(blueprint), the Hospital asks for a new product design, as the logs show the Hospital (ID = 2) is appended to the product(#0) details. Also, an event is emitted announcing that the Hospital is asking for a design offer as demonstrated in figure 6.

#### 2) Hospital is Asking for Manufacturing Offers

**FIGURE 7.**
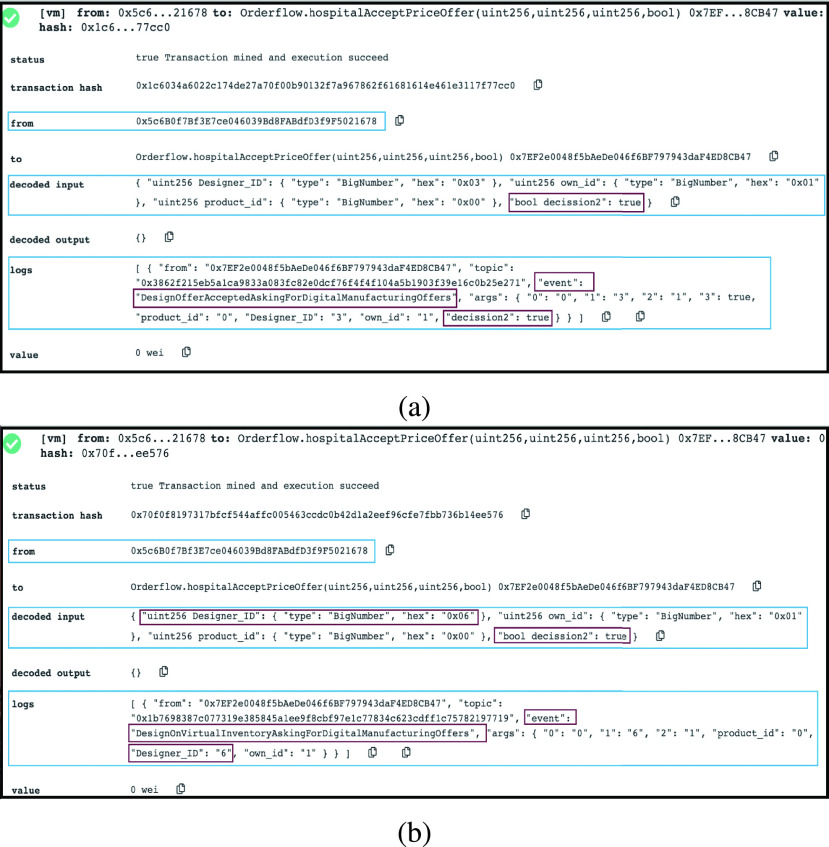
(a) Logs showing that the Hospital is asking for manufacturing offers. (b) Logs showing that the Hospital is asking for manufacturing offers for a product on the virtual inventory.

This function marks the beginning of the second phase that is the manufacturing phase. Two alternatives are projected in the logs below. When the authority and Hospital already approve a new design, the input decision is true. The event states that the design is accepted, and the Hospital requires digital manufacturing offers for it as illustrated in figure 7a. The second alternative is for the designs available on the virtual inventory, and that allows the Hospital to skip the first phase to commence the process with this function by adding the virtual inventory (ID = 6) as the designer in the required field along with the approval of design (decision = true) as shown in figure 7b. As a result, an event is triggered to announce that the design is on VI and that the Hospital asks for a digital manufacturing offer.

#### 3) Authority is Approving/Rejecting the Final Product

**FIGURE 8.**
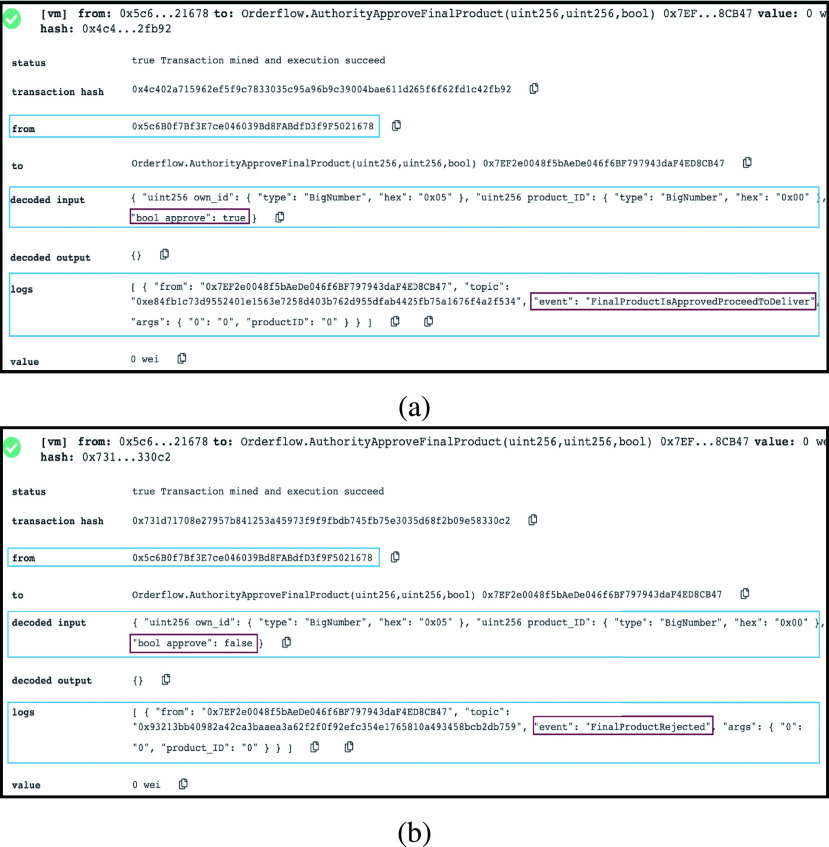
(a) Logs showing that Authority is approving the final product. (b) Logs showing that Authority is rejecting the final product.

**FIGURE 9.**
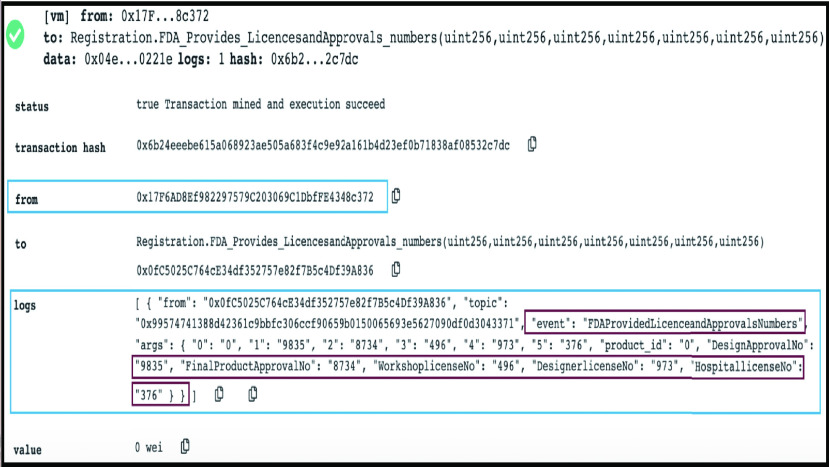
Logs showing that authority is providing licences and approvals numbers.

After the product is manufactured and IPFS hash is provided, the Authority can review all manufacturing and IoT devices’ records to accept or reject the final product. The Authority is responsible for making the decision, and as presented in the logs in figure 9, the announcement will alternate depending on the decision value input. If the decision is to accept the emitted event, the final product is approved and delivered as shown in figure 8a. While if it is false, the event declares that the final product is rejected, as shown in figure 8b.

### B. Registration Smart Contract

The Ethereum addresses of the stakeholders and the Registration SC are provided in table 2. In the following, we demonstrate the testing of registration SC functions.

#### 1) Authority is Providing Licenses and Approvals Numbers

FDA returns all the required approvals and license numbers as shown in figure 9 where all required fields are returned to MOH, and an event announces that FDA provided licenses and approvals numbers.

#### 2) MOH is Confirming Registration Completion

**FIGURE 10.**
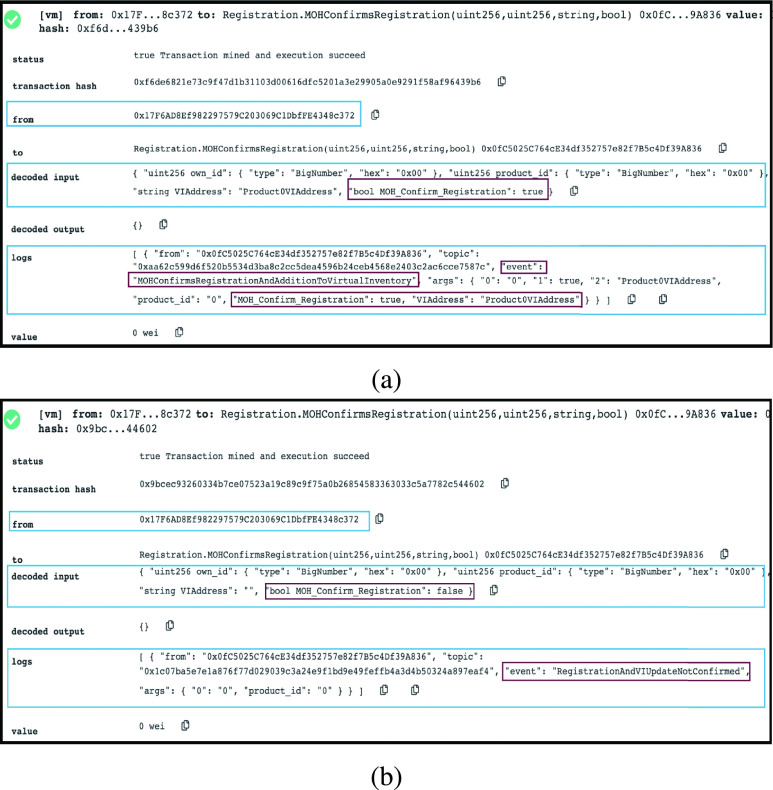
(a) Logs showing that MOH is confirming that registration is done. (b) Logs showing that MOH is confirming no registration is done.

Finally, the MOH registration entity confirms the registration completion and the addition of the design if it is new to the virtual inventory. Figures 10a and 10b show two possible scenarios. When registration is completed successfully, an agreement with the designer is completed successfully, and the new design is added to the virtual inventory, an event is triggered to announce that MOH confirms registration. The alternative is that registration or uploading a new design to virtual inventory is not completed successfully where an event announces the same.

## V. Discussion

In this section, we evaluate our proposed approach by analyzing its cost and security features. We compare our solution with the existing solutions to show its novelty. Also, we discuss our solution from the generalization point of view in terms of its applicability.

### A. Cost Analysis

We perform our testing and compilation using the Ethereum blockchain. Gas is used to pay for all transactions executed on the blockchain, and ether tokens are used to pay for gas. Each function’s net cost in the smart contracts processed on the blockchain is divided between the execution cost (the cost of the function’s execution including the storage cost and the update of parameters and variables states in the smart contracts’ costs) and the transaction gas costs that include deployment-related factors and transferring the data. Table 3 and Table 4) show the gas costs of the functions’ execution in the supply SC and registration SC respectively. The net prices converted to USD are provided as well. The gas prices used in the tables (3, 4) is the average gas (102 Gwei) prices calculated on Jan $2^{nd}$, 2021. The price source is the ETH Gas Station [36]. The Callers of the functions of the smart contract showing in Tables ((3, 4)) are the MoH, Designer, Hospital, digital manufacturing Workshop, RIs or the Certification Authority, each function’s caller is displayed in the table under the “Function’s Caller” column.

**TABLE 3:** Costs of the COVID-19 Supply SC’s Functions in Gas and USD

Function Description	Function Caller	Transaction Cost	Execution Cost	Total Gas	Cost( $\$ $)
Add new Order	MOH	46117	24525	70642	$\$ $4.19
Asking for design offer	Hospital	45225	23633	68858	$\$ $4.09
Confirming Required manufacturing details	RIs	50099	26523	76622	$\$ $4.55
Asking for design approval	Designer	70411	46579	116990	$\$ $6.95
Design approval	Authority	46522	24738	71260	$\$ $4.23
Asking for digital manufacturing offers	Hospital	33988	12012	46000	$\$ $2.73
Asking for digital manufacturing offers from VI	Hospital	29433	7457	35086	$\$ $2.08
Offering digital manufacturing	Digital workshop	66132	44348	110480	$\$ $6.56
Accepting/rejecting manufacturing offer	Hospital	28531	6555	35086	$\$ $2.08
Requesting final product approval by Authority	Workshop	50095	26455	76550	$\$ $4.54
Approving/rejecting final product	Authority	31212	9428	40640	$\$ $2.41
Confirming delivery and successful operation	Hospital	27109	5133	32242	$\$ $1.91
Confirming Registration and Virtual Inventory update	MOH	46130	24410	68540	$\$ $4.07

**TABLE 4:** Costs of the Registration SC’s Functions in Gas and USD

Function Description	Function Caller	Transaction Cost	Execution Cost	Total Gas	Cost( $\$ $)
Add new Record	MOH	24783	3255	28038	$\$ $1.66
Requesting FDA Licences and Approvals	MOH	136999	111631	248630	$\$ $14.76
Providing Licences and Approvals Numbers	Authority	129176	106304	235480	$\$ $13.98
Confirming Registration and Adding the New Design to the Virtual Inventory	MOH	51281	27961	79242	$\$ $4.70

The costs of transactions are relatively high, considering the large expected number of transactions given the wide variety of parts and participants. However, we need to emphasize that Ethereum gas fees are dependent on network usage and congestion. During the last year, movements like the DeFi (Decentralized Finance) [37] has created a boom increasing the use of the Ethereum network leading to congestion and rapidly growing gas fees. After some time, such prices will naturally go down. Also, Ethereum economic system is expected to change once the Ethereum protocol upgrade “Serenity” is out. Ethereum will shift from PoW (Proof of Work) to PoS (Proof of Stake), in which validators staking their gas will be replacing miners, and price dependency on network congestion will be limited. Meanwhile, one alternative that allows low transaction costs is the use of the private blockchain. A single organization (MOH in our proposed solution) can have access and authority to create the network in a private blockchain, making it a small-scale decentralized system. Private blockchain allows transaction fees to be minimal, independent of the number of requests, faster, and at higher efficiency. Another alternative is using emerging lower-cost Ethereum networks, such as zkSync, that allow low costs on Ethereum. ZkSync allows fees below $\$ $.01 per transaction [37]. Overall, we can conclude that our proposed solution is cost-efficient.

### B. Security Analysis

We discuss the security aspects of our blockchain-based solution. Blockchain technology features allow trust, immutability, and traceability, which in return enables achieving some of the main security targets, including accountability, authorization, integrity, and non-repudiation. Our proposed solution is resilient against some known attacks, such as the man-in-middle attack (MITM) and the replay attack. The main security issues are further clarified as follows:

#### 1) MITM and Replay Attacks

In the blockchain, each participant has a private and a public key. The private key is used to cryptographically signing each transaction. This hinders any external intrusion and prevents an attacker from changing any transaction without the private key. Thus, the proposed solution is secured enough against the MITM attack. Furthermore, duplicate transactions are rejected and discarded by mining nodes. This prevents any form of replay attacks. Furthermore, attackers trying to call functions repetitively to make the network congested are limited as attackers running a function spend gas fees every time they run it. As a result, gas fees deductions will limit and prevent repetitive attacks because the attacker will run out of Ether gas at some point.

#### 2) Accountability

Each participant is identified by its Ethereum address used to ensure accountability. Ethereum addresses are used to trace back each participant’s transactions.

#### 3) Non-Repudiation

All transactions are signed cryptographically using participants’ private keys, and all logs are saved in a tamper-proof manner, which hinders anyone from denying their actions on the blockchain.

#### 4) Authorization

In the smart contract functions, the caller is always checked before using the function to ensure that only specific participants are allowed to execute certain functions, thereby enabling proper authorization for each participant depending on their specific roles in the network.

#### 5) Integrity

Our proposed blockchain-based solution ensures the integrity of all transactions, events, and logs to enable reliable traceability for the product from the designer to the manufacturer and finally till it reaches the hospital. The transactions’ history and data are stored securely on chain logs. Moreover, the large-size data files are stored on the IPFS, and only the hashes are stored on the chain.

### C. Comparison With the Existing Solution

We compare our proposed decentralized digital manufacturing and supply chain system with the existing solutions as shown in Table 5. The existing solutions are based on the traditional manufacturing and supply chain systems, where products are manufactured in a central location then distributed worldwide. However, our proposed solution enables decentralized manufacturing of medical devices and ensures reliable and traceable supply chain management. Also, higher production flexibility and product customization are allowed in our digital manufacturing proposed solution; digital manufacturing methods like 3D printing and CNC machining allow customization to products much more than traditional manufacturing, where production lines are fixed. In contrast, in digital manufacturing, the same batch can include various products, shapes, and sizes. This allows a much higher flexibility in digital manufacturing than traditional, where customizing a product or shifting production lines from one product to another is inflexible, costly, and time-consuming.

**TABLE 5:** Comparison Between Proposed and the Existing Manufacturing and Supply Chain Solutions

Comparison Criteria	Traditional manufacturing and supply chain systems	[26]	[27]	Our proposed solution
Decentralized Production	No	No	No	Yes
Data Privacy Protection	No	Yes	Yes	Yes
Data Tamper Resistance	No	Yes	Yes	Yes
Product Customization	No	No	No	Yes
Production Flexibility	No	No	No	Yes
Cost of Manufacturing Complex Parts	High	High	High	Same as simple parts
Cost of Manufacturing Mass Produced Parts	Low	Low	Low	High
Lead Time	Long	Long	Long	Short
Material Waste	High	Low	High	Low
$CO_{2}$ Emissions	High	High	High	Low

Furthermore, our proposed solution ensures secured product and data traceability throughout the entire process and among all participating stakeholders. Blockchain technology is distinguished by trust, privacy protection, and traceability benefits because of private keys and cryptographic signatures. Also, blockchain ensures the data stored on the chain is tamper-proof and resistant against any attack because of its structure where blocks build on each other, and any change in any transaction can be monitored instantly.

Digital manufacturing allows producing complex products and grouping parts, eliminating the assembly phase at the exact cost of simple parts. For simple parts that are mass-produced, the costs in traditional manufacturing are less compared to digital manufacturing [38]. However, most of the cost is related to the digital machines (3d-printers and CNC machines), which is expected to decrease with economies of scale as rapid technological advancements and broader adoption and spread co-occur. Moreover, the decentralized nature of our proposed solution gives it the advantage of much faster lead times compared to traditional manufacturing. Specifically, during the COVID-19 pandemic considering the borders closure, the ban of the movement worldwide, and the strict and elongated procedures at borders. Finally, the proposed digital manufacturing and supply system minimizes wasted material, reduces $CO_{2}$ emissions, and is considered a much more environment-friendly system than the existing solutions.

We conclude that our proposed system is superior to the other systems considering cost, time, security, efficiency, environmental, and sustainability aspects. In addition, our proposed solution is more flexible, efficient, and reliable than the traditional manufacturing and supply management systems.

### D. Generalization

Our proposed blockchain-based decentralized digital manufacturing solution can be adapted into various applications other than the medical devices and supplies for COVID-19 as described below:

1) This work can be customized as per the need of other critical sectors in the country that may suffer from the closure of the border, extensive and lengthy procedures, and extended lead times to deliver necessary products and supplies, including the energy sector, educational sector, and others.
2) Unexpected and catastrophic events originate sudden peak demands and require fast and flexible production and supply systems. Including human-made wars or natural disasters like earthquakes, tsunamis, volcano eruptions, or tornadoes that result in massive destruction and require a fast and flexible supply of required products and parts for fast and efficient rebuilding and renovating the hurt cities or countries.
3) Enterprises aim to shift to decentralized manufacturing by establishing their distribution network of digital manufacturing workshops, providing more flexible and agile services for their customers worldwide, and reducing inventory and transportation costs.

## VI. Conclusion

This paper has proposed a blockchain-based solution to automate the decentralized digital manufacturing for COVID-19 medical supplies in a transparent, traceable, reliable, auditable, secure, and trustworthy manner. Also, the proposed solution aims to overcome certain challenges posed by the traditional supply chain systems due to the lack of secured traceability of medical supplies. We developed two smart contracts and proposed fourteen algorithms to implement the full functionalities and trigger events and notifications related to the manufacturing of COVID-19 medical supplies. We incorporated the decentralized storage of IPFS into the Ethereum blockchain to securely manage large-size data related to the manufacturing of COVID-19 medical supplies. The proposed solution can assist in ensuring the authenticity of digitally manufactured COVID-19 medical supplies and the attestation and certification of a digitally manufactured product in geographically dispersed digital manufacturing workshops and designer entities. We evaluated the effectiveness of the proposed approach using different parameters, such as cost, security, and comparing it with the existing solutions. The evaluation results show that our solution is cost-efficient, secure against well-known attacks and vulnerabilities, and has advantages over existing traditional solutions. The proposed solution is generic and can be applied in similar crises, events, and situations with minimal efforts and modifications. Limitations of our work include the digital manufacturing spread and adoption as the technologies are still maturing. However, adoption rates are continuously increasing with the rapid technological advancements. Also, some other limitations include governance issues like assigning responsibilities, policy setting, updates, and upgrades of smart contracts. In the future, we aim to deploy and test our solution on the real Ethereum network and build an end-to-end DApp for different stakeholders.
